# Elucidating the difference between mind-wandering and day-dreaming terms

**DOI:** 10.1038/s41598-024-62383-7

**Published:** 2024-05-21

**Authors:** Hagar Shimoni, Vadim Axelrod

**Affiliations:** The Gonda Multidisciplinary Brain Research Center, Bar Ilan University, 5290002 Ramat Gan, Israel

**Keywords:** Human behaviour, Consciousness, Cognitive neuroscience

## Abstract

Self-generated thoughts have been widely investigated in recent years, while the terms “mind-wandering” and “day-dreaming” are usually used interchangeably. But are these terms equivalent? To test this, online study participants were presented with situations of a protagonist engaged in self-generated thoughts. The scenarios differed with regard to type of situation, the activity in which the protagonist was engaged in, and the properties of the self-generated thoughts. Two different groups evaluated the same situations; one group evaluated the extent to which the protagonist mind-wandered and another the extent to which the protagonist day-dreamt. Our key findings were that the situations were perceived differently with regard to mind-wandering and day-dreaming, depending on whether self-generated thoughts occurred when the protagonist was busy with another activity and the type of self-generated thoughts. In particular, while planning, worrying, and ruminating thoughts were perceived more as mind-wandering in situations involving another activity/task, the situations without another activity/task involving recalling past events and fantasizing thoughts were perceived more as day-dreaming. In the additional experiment, we investigated laypeople’s reasons for classifying the situation as mind-wandering or day-dreaming. Our results altogether indicate that mind-wandering and day-dreaming might not be fully equivalent terms.

## Introduction

Most cognitive research to date has been dedicated to understanding task- and goal-based behavior, but our lives are not limited only to performing task X or Y as a substantial proportion of our daytime is devoted to self-generated thoughts^1–3^. Self-generated thinking (or mind-wandering/day-dreaming; see below) is a highly multifaceted phenomenon. Self-generated thoughts can be a distraction from the main activity (i.e., task-unrelated thoughts^4,5^), but can also occur when a person is not engaged in any type of activity, as in a downtime period^6^. Self-generated thoughts also vary substantially by type, including thoughts about the past such as autobiographical memories^7–13^, fantasizing^3,14–16^, future planning^8,9,17–20^, worrying and ruminative repetitive thoughts^21–24^. Self-generated thoughts can also be intentional or unintentional^25^.

**Figure 1:**
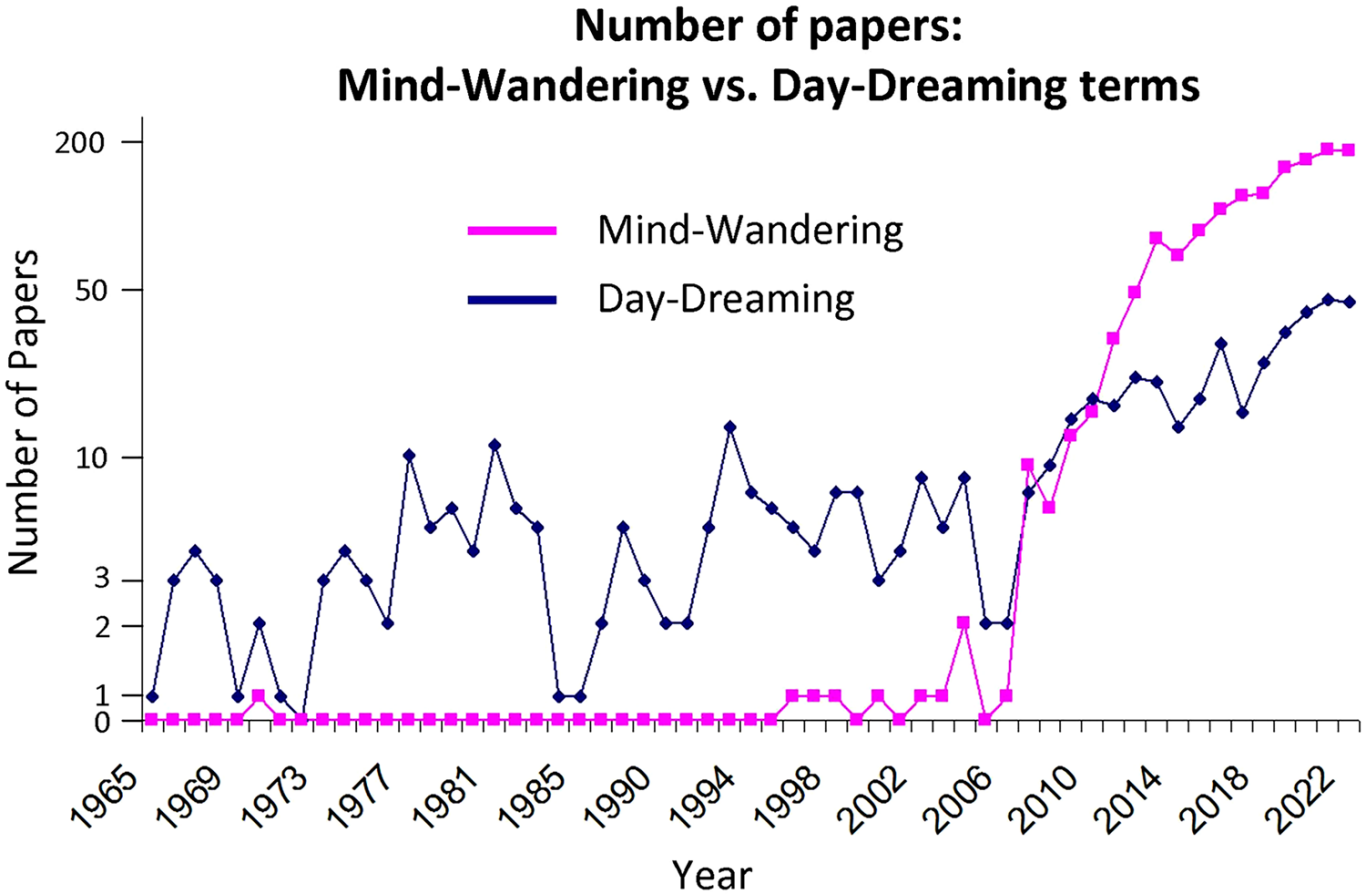
Number of papers that used “mind-wandering” or “day-dreaming” terms. The search was conducted with the Pubmed database across abstracts/titles using the keywords “mind-wandering”, “mind wandering” or “mindwandering” (mind-wandering query) and “day-dreaming”, “day dreaming” and “daydreaming” (day-dreaming query). The Y axis is a logarithmic scale. Note that until mid-2000, the term “mind-wandering” was almost never used.

From a terminological point of view, for several decades until about the mid-2000s, self-generated thoughts were predominantly referred to in scientific literature as “day-dreaming” (Fig. 1). Over the last decade-and-a-half, there has been a surge in self-generated thinking exploration^26,27^ while the term “mind-wandering” became more dominant (Fig. 1). Therefore, contemporary mind-wandering research can be viewed to a great extent as a continuation of earlier day-dreaming research^28^. To date, except for the specific pathological condition of maladaptive day-dreaming^15,16,29^, many studies have used the terms “day-dreaming” and “mind-wandering” interchangeably^5,6,10,27,30–34^. But are “day-dreaming” and “mind-wandering” terms really equivalent? This question has puzzled researchers previously, for example Kalina Christoff wondered “Is mind wandering the same as daydreaming? These questions remain unanswered.”^35^. Philosopher Fabian Dorsch (see Ref.^36^) offered a theoretical proposal suggesting that while mind-wandering is a spontaneous stream of thoughts without a specific focus and relatively lower intentionality, focused day-dreaming is a focused (e.g., on a specific topic) stream of thought with relatively greater intentionality. Strikingly, we are not aware of any study that has tested empirically whether the terms “day-dreaming” and “mind-wandering” are perceived equivalently. Answering this question is important from both theoretical and practical perspectives. From a theoretical point of view, it is obviously important to use the most precise terminology. From a practical point of view, while some questionnaires like the Daydreaming Frequency Scale^37^ or NEO Personality Inventory^38^ use the term “day-dreaming,” other questionnaires like the Mind-Wandering Questionnaire^39^ and the Deliberate and Spontaneous Mind-wandering Scale^40^ use the term “mind-wandering.” However, if day-dreaming and mind-wandering are not fully equivalent terms, merely using these different terms in the questionnaires might bias the results and complicate their interpretation when comparing or correlating them from different questionnaires^39^.

It is noteworthy, that irrespective of potential differences between “day-dreaming” and “mind-wandering” terms, terminology and conceptual definitions in the field of self-generated processing and mind-wandering have been debated. For example, it has been suggested that the generic term “mind-wandering” might conflate different phenomena^41–44^, thus complicating interpretation of findings and theoretical development. Also, in many studies, mind-wandering is defined as task-unrelated thoughts (TUT)^5^ or stimulus-independent thoughts (SUT)^45^, but some oppose equating mind-wandering to TUT and/or SUT because this definition does not take into account important aspects of mind-wandering such as its dynamics^46–48^. Critically, the inquiry of the present paper is different and unrelated as to whether mind-wandering and day-dreaming should (or should not) be equated to TUT or SUT. Here, we asked to what extent mind-wandering and day-dreaming are equivalent and perceived similarly.

To test whether “mind-wandering” and “day-dreaming” terms are perceived similarly, for our online study we composed a questionnaire in which each item included a depiction of a situation with a protagonist engaged in self-generated thoughts. Across two experiments, the scenarios differed with regard to type of situation (i.e., whether the protagonist was engaged in the main activity or had a downtime), activity the protagonist was engaged in (i.e., relatively passive engagement such as attending a meeting or the activity with relatively active engagement such doing homework), type of self-generated thoughts, intentionality of self-generated thoughts and duration of self-generated thoughts. Critically, the same questionnaire was presented to two groups of participants to evaluate the level at which the protagonist was mind-wandering (Group 1) or day-dreaming (Group 2). In our final experiment, we investigated laypeople’s reasons for classifying the situation as mind-wandering or day-dreaming.

## Methods

### Participants

The study was conducted using the Amazon Mechanical Turk crowd-sourcing service (https://www.mturk.com/). A total of 882 participants participated in three sets of experiments: (1) The main experiments 1 and 2 in which participants made judgments about the situation with regard to mind-wandering (MW) and day-dreaming (DD); (2) The replication experiments 1 and 2 in which we replicated the results of main experiments 1 and 2; (3) The experiment in which we investigated why situations with some self-generated thoughts were classified more as “mind-wandering” while others were classified as “day-dreaming”. The participants were recruited in the USA, and all spoke English as their first language. The study was approved by the Interdisciplinary Studies Unit ethics committee at Bar-Ilan University (Israel), and all stages of the study were conducted in full accordance with this approval. The study was conducted online, and the acquired data were anonymous. Participants provided their informed consent by marking the checkbox.

Each of the two main experiments included two groups of participants: one making judgments about the situation with regard to MW and another making judgments about the situation with regard to DD. For participation, the participants received monetary compensation ($1.7 in Experiment 1 and $2.5 in Experiment 2). It took participants about 10 to 15 min to complete the experiment. A total number of 464 participants took part in two experiments (228 in experiment 1 and 206 in experiment 2). We determined the sample size a priori (i.e., before the main experiment) by conducting a preliminary pilot (26 and 25 participants in the mind-wandering and day-dreaming groups, respectively). We found that the difference between the mind-wandering and day-dreaming groups (two-sample, two-tail *t*-test) reached Cohen's d value of 0.5. Accordingly, using G*Power 3 tool^49^, we estimated that to achieve Cohen's d = 0.5 effect size (power = 0.8), the number of participants per group (after exclusion, see below) should be about 60. To ensure high data quality, following recommendations^50,51^ a number of participants were excluded (the details of the exclusion procedure are explained in the data analysis section). The final dataset after exclusion was as follows. 131 participants in Experiment 1 (MW group: 64 participants, mean age 36.14, age standard deviation 10.68, 28 females, and 5 left-handed people; DD group: 67 participants, mean age 38.6, age standard deviation 10.94, 29 females, and 8 left-handed people). 130 participants in Experiment 2 (MW group: 64 participants, mean age 35.56, age standard deviation 8.99, 31 females, and 1 left-handed people; DD group: 66 participants, mean age 38.7, age standard deviation 11.49, 30 females, and 6 left-handed people). In Experiment 2, the average age of the participants was slightly higher in the DD group than in the MW group. In a control analysis, we equalized age between the two groups by excluding the five oldest participants from the DD group. The results obtained from the control analysis were very similar to those reported in the main text. In addition, we conducted a control analysis by including participants’ ages as a regressor (see “Results”).

To ensure replicability of our results, we repeated Experiments 1 and 2. For participation, the participants received monetary compensation ($1.7 in Experiment 1 and $2.5 in Experiment 2). It took participants about 10 to 15 min to complete the experiment. A total number of 333 participants took part in two replication experiments (169 in Experiment 1 and 164 in Experiment 2). After participants’ exclusion, the final dataset was as follows. There were 124 participants in Experiment 1 (MW group: 61 participants, mean age 41.23, age standard deviation 10.95, 25 females, and 6 left-handed; DD group: 63 participants, mean age 43.11, age standard deviation 13.27, 29 females, and 7 left-handed). There were 129 participants in Experiment 2 (MW group: 67 participants, mean age 41.71, age standard deviation 11.37, 28 females, and 2 left-handed people; DD group: 62 participants, mean age 43.55, age standard deviation 13.18, 28 females, and 1 left-handed).

To investigate why situations with some self-generated thoughts were classified more as “mind-wandering” while others were classified as “day-dreaming,” we conducted an additional experiment. The participants received monetary compensation of $1.50, and the experiment took up to 10 min to complete. First, we conducted a pilot experiment (15 participants, mean age 46.6, age standard deviation 13.1, 8 females, and all right-handed) in which the participants wrote their reasons in open form as free text. Then, we conducted the main experiment, in which the questions were presented in a closed form as multiple-choice items. A total of 70 participants took part in the main experiment. Eleven participants were excluded due to providing answers inconsistent with those on the “mind-wandering”/“day-dreaming” preference question, and one participant was excluded because of not speaking English as their first language. The final sample size included 58 participants (mean age 45.18, age standard deviation 11.25, 27 females, and four left-handed).

### Experimental design

The questionnaire items included a depiction of a scenario about a person (for a similar design, see the recent paper by Irving and colleagues^44^). For example: “A person has been busy with a task (e.g., doing homework), but at some point started to fantasize about something unrelated (e.g., about a romantic date tomorrow)”. Both MW and DD groups received identical situation descriptions. Critically, the question that followed the description of a situation differed between the two groups: While the MW group was asked “To what extent do you think the person mind-wandered?”, the DD group was asked “To what extent do you think the person day-dreamt?” The answers were given using a Likert scale: 1 (minimal extent)–5 (maximal extent). To ensure the quality of the online data^50–53^, our questionnaire also included five “catch” questions (also known as “bogus” items) of different types. In particular, we included two elementary arithmetic questions (i.e., “Calculate 17–12” and “Calculate: 37–36”), two common-sense questions formulated intuitively (i.e., “A runner just completed a marathon (42 km) in extremely hot weather. To what extent is he tired?” and “An athlete got injured just before an important competition. To what extent he is disappointed?”) and one common-sense question formulated in a counterintuitive way (i.e., “A student just found out that he failed an extremely important exam. To what extent do you think he is pleased with this situation?”). The arithmetic questions had 1–5 possible answers, and the common-sense questions were given using a Likert scale: 1 (minimal extent)–5 (maximal extent). The added value of the common-sense question was that on the one hand, similar to the items of interest, they depicted the situation, but on the other hand, these situations had correct answers that could be verified. The order of the items in the questionnaire, including the “catch question” items, was pseudo-random (in a different order for each participant). A similar experimental design was used in both experiments (see below).

The experimental design of Experiment 1 is shown in the figure in the “Results” section of Experiment 1. The situations in this experiment depicted a person engaging in two possible types of activity (i.e., attending a meeting/lecture/class or being busy with a task, like doing homework) or no activity (i.e., downtime). We included two types of activity to test whether there is a difference between relatively passive engagement (i.e., attending a meeting/lecture/class) and the activity with relatively more active engagement (i.e., doing homework). The situations also varied with regard to the type of self-generated thought of the protagonist (i.e., recalling past events, fantasizing, planning, worrying, and ruminating). For instance, a situation in which a person attended a meeting/lecture/class (i.e., a type of activity) while they were recalling past events (i.e., the type of self-generated thought) was as follows: “A person has been attending an important meeting, lecture, or class, but at some point started to recall something unrelated from the past (e.g., yesterday's romantic date).” The situations followed exactly the same pattern, while only the specification of activity and self-generated thought varied. As described above, the question that followed the description of the situation differed between the two groups: the MW group was asked “To what extent do you think the person mind-wandered?”, and the DD group was asked “To what extent do you think the person day-dreamt?” The total number of items (i.e., situations) was fifteen. For the full list of situations for Experiment 1, see Supplementary Table 1.

The experimental design of Experiment 2 is shown in the figure in the “Results” section of Experiment 2. The situations depicted a person attending a meeting/lecture/class. The situations varied with regard to intentionality of the self-generated thought (i.e., with or without intention) and duration (i.e., from several seconds [short] to 20 min [long]). For example, the situation in which a person was engaged for a short time in unintentional fantasizing was as follows: “A person has been attending an important meeting, lecture or class. At some point, just for several seconds, he/she without intention disengaged from the event by fantasizing about something unrelated (e.g., about a romantic date tomorrow).” The situations followed exactly the same pattern, while the specific self-generated thought, duration, and intention varied. The total number of items (i.e., situations) was twenty. For the full list of situations, see Supplementary Table 2.

The experiment examining why situations with some self-generated thoughts were classified more as mind-wandering while others were classified more as day-dreaming was conducted in two stages. First, in a pilot experiment, the participants were presented with five situations from Experiment 1: “planning,” “worrying,” and “ruminating” during another activity and “recalling past episodes” and “fantasizing” without another activity (items 3, 4, 5, 11, and 12 in Supplementary Table 1). After description of the situation, half the participants were asked “Would you be more likely to say that this person mind-wandered than day-dreamed?” (Yes/No answers), and another half were asked: “Would you be more likely to say that this person day-dreamed than mind-wandered?” (Yes/No answers). For each of five questions, there was a follow-up question asking the participants to provide reasons for their choices in a free format. The participants were asked to provide reasons only if they choose mind-wandering over day-dreaming for planning, worrying, and ruminating, and day-dreaming over mind-wandering for recalling past episodes and fantasizing. The reasons provided in the free text form in the pilot experiment were used to construct the multiple-choice items in the closed-form version. So, in the main experiment we used the same design as the pilot experiment, with the only difference being that participants selected the reasons from a provided list from which more than one reason could be selected.

### Data analysis

The data were analyzed using MATLAB homemade script^54^ and the JASP statistical package. To ensure quality of the online data^51^, we excluded the data of participants who answered incorrectly to the “catch” questions. Specifically, we excluded from the analysis those participants who answered the arithmetic questions incorrectly (i.e., a single answer was accepted) or answered “1” or “2”, or “3” to the common-sense questions formulated intuitively (e.g., we excluded participants who answered that a runner was not so tired after completing a marathon in extremely hot weather). This procedure resulted in excluding about 40% of participants (see section “Participants”). We obtained qualitatively similar results with more and less stringent criteria for participant exclusion. In a more stringent scenario, in addition to the criteria described above, we also excluded those participants who gave incorrect answers to the common-sense questions formulated in a counterintuitive way (exclusion rate of about 70%). In a less stringent scenario, we excluded those participants who answered the arithmetic questions incorrectly or answered “1” or “2” to the common-sense questions formulated intuitively (exclusion rate of about 25%). The main goal of our statistical analyses was to examine the difference between the MW and DD groups. For that, we conducted mixed repeated-measures ANOVA with factors of interest (e.g., “type of activity” in Experiment 1) as the within-subject factor and group of participants (MW or DD) as the between-subject factor. The differences were evaluated further using post-hoc two-sample two-tailed *t*-tests. Bonferroni multiple-comparison correction was applied where needed.

## Results

### Experiment 1

**Figure 2:**
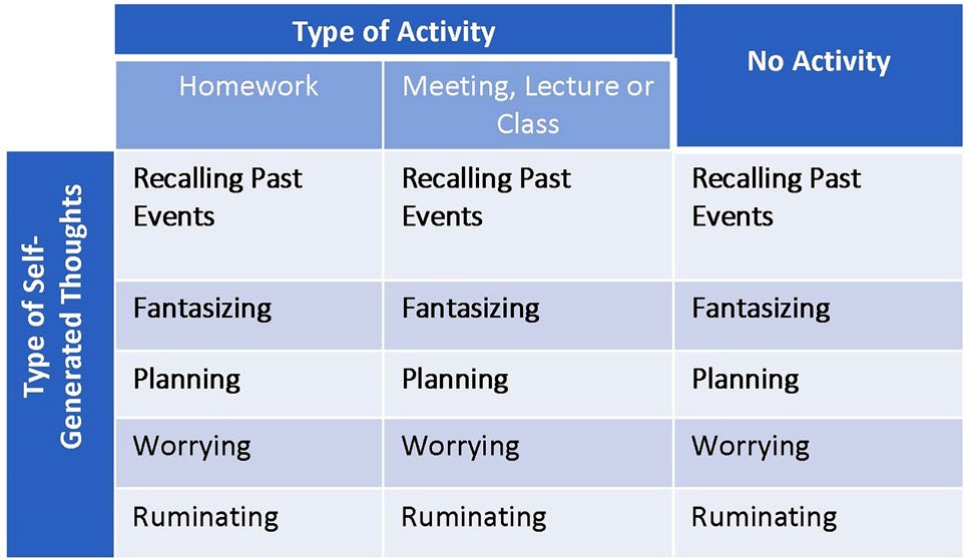
Experimental design of Experiment 1. The design included situations in which a protagonist was engaged in two types of activities or no specific activity (i.e., the columns in the diagram). The situations also varied with regard to the type of self-generated thought of the protagonist (i.e., the rows in the diagram). Exactly the same situations were presented to two groups of participants, who were asked to rate (Likert scale rating: 1–5) to what extent the protagonist mind-wandered (MW group) or day-dreamt (DD group).

The design of the experiment is shown in Fig. 2. The design included situations in which the protagonist was engaged in two types of activity (i.e., attending a meeting/lecture/class or being busy with a task, like doing homework) or no activity (i.e. downtime, like waiting in a queue). The situations varied with regard to the type of self-generated thought (i.e., recalling past events, fantasizing, planning, worrying, and ruminating). Exactly the same situations were presented to two groups of participants who were asked to rate (Likert scale: 1–5) to what extent the protagonist mind-wandered (MW group) or day-dreamt (DD group).

**Figure 3:**
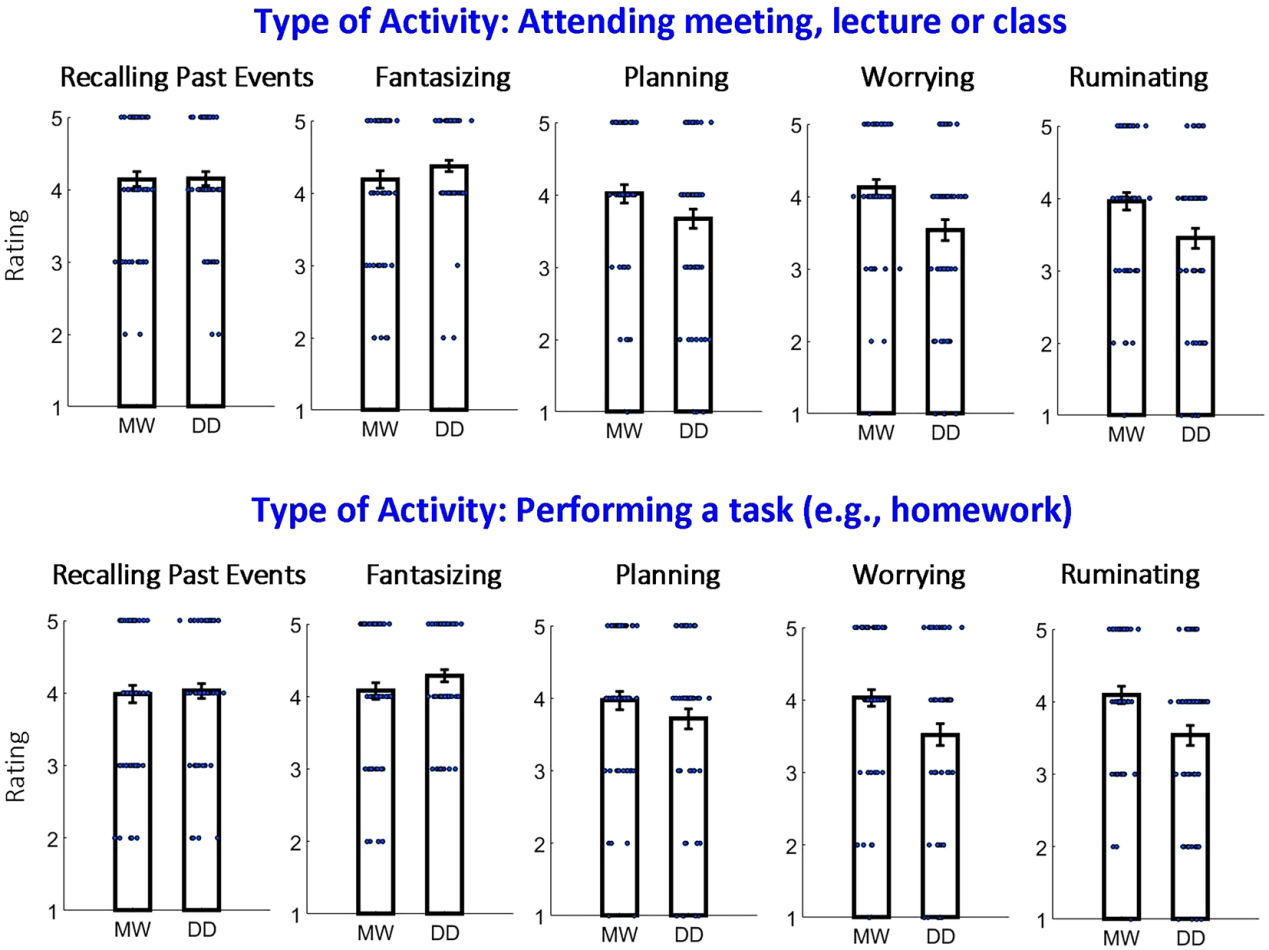
Results of Experiment 1 for situations with two types of activities: attending a meeting/lecture/class or being busy with a task like doing homework. The small blue dots represent the results of individual participants. The error bars reflect the standard error of the mean. MW (mind-wandering) and DD (day-dreaming) represent two groups of participants. Note that the two types of activity were similar with regard to the relationship between mind-wandering and day-dreaming.

First, in our analysis, we focused on the situations in which a protagonist engaged in two types of activity (i.e., attending a meeting/lecture/class or being busy with a task, like doing homework). These results are shown in Fig. 3. With regard to perceiving mind-wandering and day-dreaming, the patterns of the results of the two activities were similar. To examine the results statistically, we conducted a mixed three-way ANOVA with “the type of question: mind-wandering (MW) or day-dreaming (DD) as the between-subject factor” and “type of activity” (two levels: attending a lecture/meeting/class or doing a task like homework) as well as “type of self-generated thought” (five levels: recalling past events, fantasizing, planning, worrying, and ruminating) as within-subject factors. Confirming the qualitative observations, there was no significant two-way interaction between “the type of question: MW or DD” and “type of activity” [F(1,129) < 1]. Since there was no difference between the types of activity (i.e., attending a meeting/lecture/class and engaging in a task like homework) with regard to mind-wandering and day-dreaming, we collapsed the data into one category (i.e., “Activity”).

**Figure 4:**
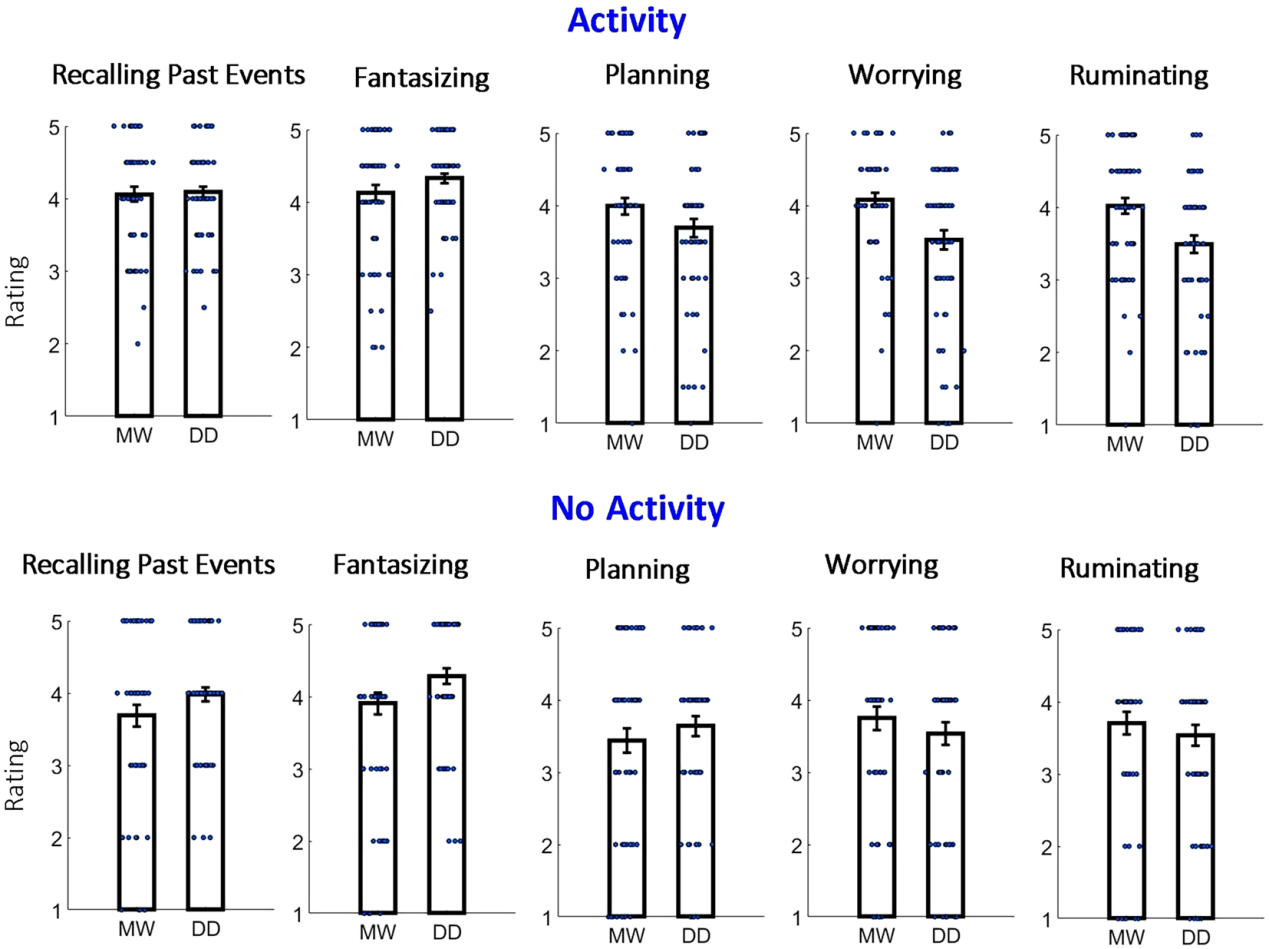
Results of Experiment 1 for situations with “Activity” (after collapsing attending a meeting/lecture/class or being busy with a task like doing homework) and “No Activity” (i.e., downtime). The same conventions as in Fig. 3 are used. Note that the relationship between mind-wandering and day-dreaming differed between the “Activity” and “No Activity” situations, suggesting that whether a protagonist in the situation was engaged in the activity influenced the way self-generated thought was perceived as mind-wandering or day-dreaming. In addition, the relationship between mind-wandering and day-dreaming differed between self-generated types of thought, suggesting that the type of self-generated thought of a protagonist influenced whether the mental activity was considered mind-wandering or day-dreaming.

The results of the “Activity” and “No Activity” (i.e., downtime) situations across five types of self-generated thought are shown in Fig. 4. To examine the effects, we conducted a mixed three-way repeated-measures ANOVA with “the type of question: MW or DD” as the between-subject factor and “activity: yes/no” as well as “type of self-generated thought” as within-subject factors. We found a significant interaction between “the type of question: MW or DD” and “activity yes/no” [F(1,129) = 6.49, p = 0.012, η^2^ = 0.006], suggesting that whether a protagonist in the situation was engaged in the activity influenced whether self-generated thought was perceived as mind-wandering or day-dreaming. Additionally, we found a significant interaction between “the type of question: MW or DD” and “type of self-generated thought” [F(4,516) = 7.205, p < 0.001, η^2^ = 0.016], suggesting that the type of self-generated thought influenced whether the protagonist's mental activity was considered mind-wandering or day-dreaming.

Given that the presence of activity influenced whether self-generated thought was perceived as mind-wandering or day-dreaming, we conducted two separate analyses for “Activity” and “No Activity.” For the “Activity,” two-way repeated-measure ANOVAs with “the type of question: MW or DD” as the between-subject factor and “the type of self-generated thought” as the within-subject factor revealed significant main effects of “the type of question: MW or DD” [F(1,129) = 4.56, p = 0.035, η^2^ = 0.017] and “the type of self-generated thought” [F(4,516) = 11.75, p < 0.001, η^2^ = 0.041], as well as significant interaction between “the type of question: MW or DD” and “the type of self-generated thought” [F(4,516) = 7.95, p < 0.001, η^2^ = 0.028]. The post-hoc *t*-test comparing mind-wandering and day-dreaming ratings revealed significantly higher mind-wandering ratings (after multiple comparison correction, n = 5; alpha = 0.05/5 = 0.01) in “worrying” [t(129) = 3.29; p = 0.001; Cohen's d = 0.58] and “rumination” [t(129) = 3.25; p = 0.001; Cohen's d = 0.57]. Slightly higher mind-wandering ratings (without reaching the significance) were also in “planning” [t(129) = 1.8; p = 0.075; Cohen's d = 0.31].

For the “No Activity,” two-way repeated-measures ANOVAs with “the type of question: MW or DD” as the between-subject factor and “the type of self-generated thought” as the within-subject factor did not reveal significant main effects of “the type of question: MW or DD” [F(1,128) < 1], revealed a significant main effect of “the type of self-generated thought” [F(4,516) = 9.92, p < 0.001, η^2^ = 0.029], as well as a significant interaction between “the type of question: MW or DD” and “the type of self-generated thought” [F(4,516) = 3.86, p = 0.008, η^2^ = 0.011]. The post-hoc *t*-test comparing day-dreaming and mind-wandering ratings revealed higher (but without reaching significance after the multiple comparison correction) day-dreaming ratings in “fantasizing” [t(129) = 2.04; p = 0.044; Cohen's d = 0.35] and also slightly higher day-dreaming ratings in “recalling past events” [t(129) = 1.69; p = 0.093; Cohen's d = 0.29].

### Experiment 2

**Figure 5:**
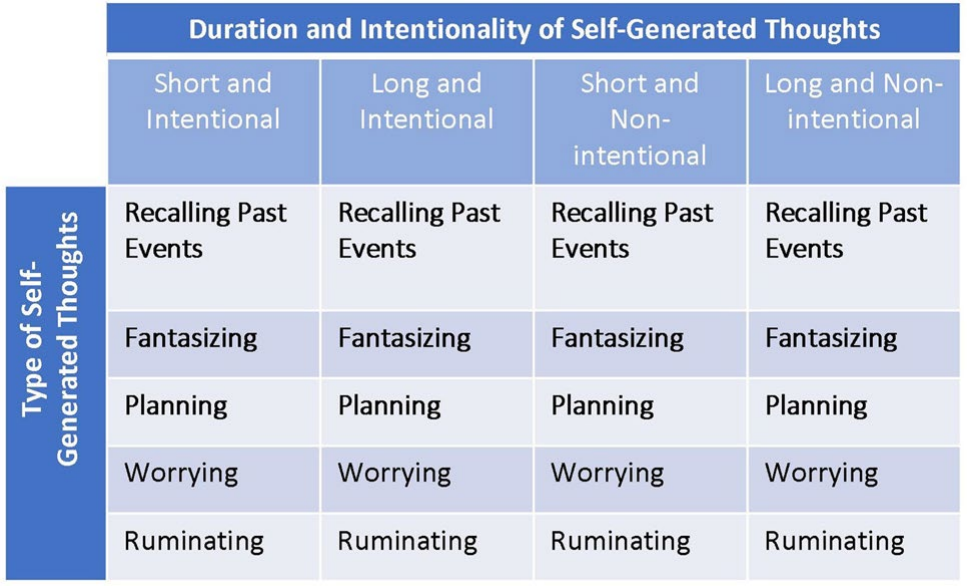
Experimental design of Experiment 2. The design included situations in which a protagonist was engaged in either “short” or “long”, as well as “with intention” or “without intention” self-generated thought (i.e., the columns in the diagram). The situations also varied with regard to the type of self-generated thought of the protagonist (i.e., the rows in the diagram). Exactly the same situations were presented to two groups of participants who were asked (Likert scale rating: 1–5) to what extent the protagonist mind-wandered (MW group) or day-dreamt (DD group).

In this follow-up experiment with a design similar to Experiment 1, we addressed two additional questions that could not be answered in Experiment 1. First, to what extent does intentionality of the self-generated thought (i.e., whether the thought was with or without intention) influences whether the protagonist was perceived as mind-wandering or day-dreaming? Second, to what extent does the duration of the self-generated thought influence whether the protagonist was perceived as mind-wandering or day-dreaming? Two groups of new participants (MW and DD groups) took part in this experiment. The design of our experiment is shown in Fig. 5. The design included only situations of attending a meeting/lecture/class, while the situations varied with regard to intentionality of the self-generated thought (i.e., with or without intention) and duration (i.e., several seconds [short] or 20 min [long]).

**Figure 6:**
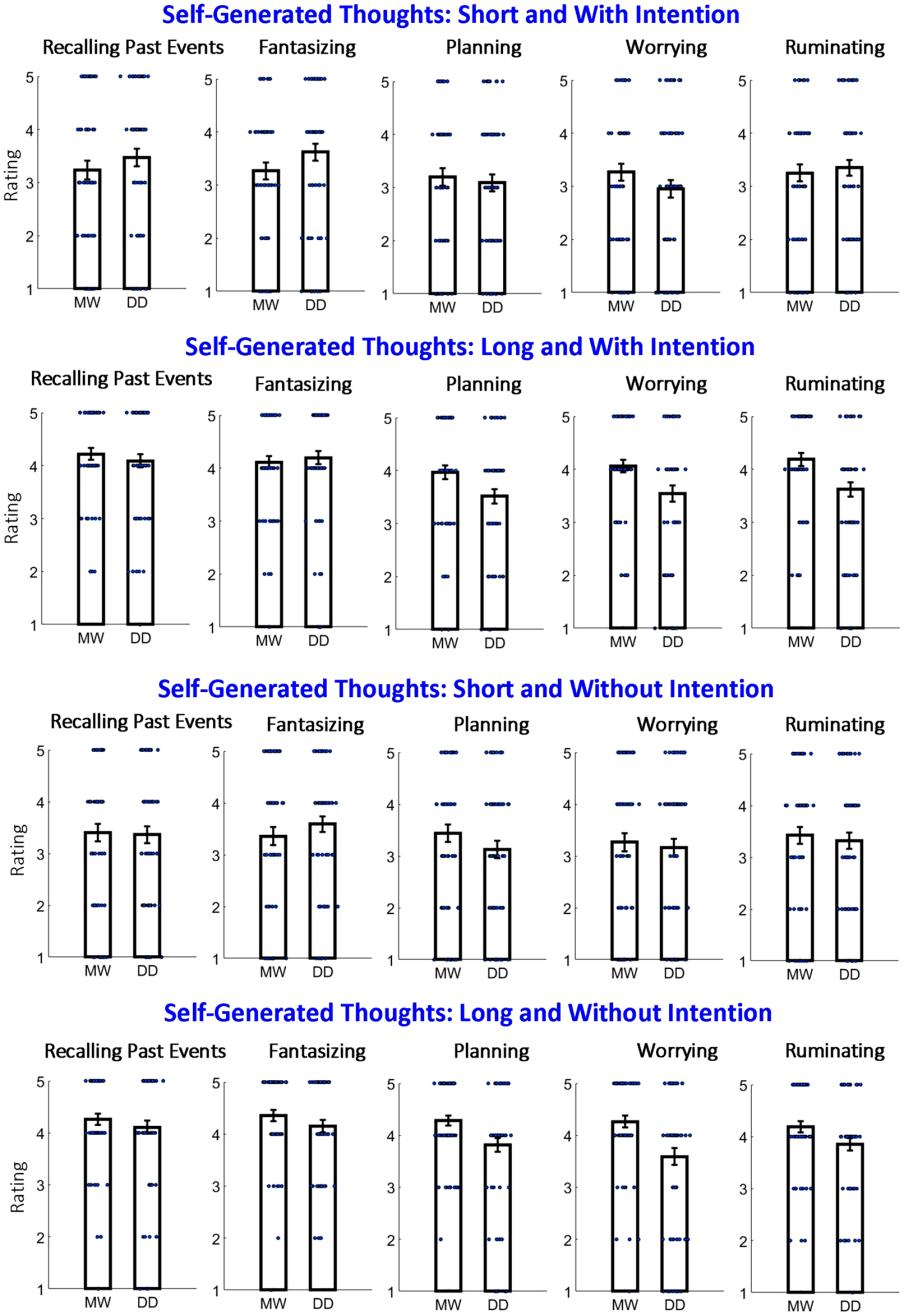
Results of Experiment 2 for situations of either “short” or “long” and “with intention” or “without intention” internally directed thoughts. The same convention are used as in Figs. 3 and 4. Note that for a given duration parameter (i.e., “short” or “long”), there was no difference between “with intention” and “without intention” with regard to the relationship between mind-wandering and day-dreaming (Row 1 is similar to Row 3; Row 2 is similar to Row 4).

The results for all conditions are shown in Fig. 6. First, we were interested in whether the intentionality of the self-generated thought influenced whether the protagonist was perceived as mind-wandering or day-dreaming. With regard to perceiving mind-wandering and day-dreaming, the patterns of results with and without intention were similar. That is, for a given duration parameter (i.e., short or long), the results of “with intention” and “without intention” were similar with regard to the relationship between mind-wandering and day-dreaming (i.e., Fig. 6: Row 1 is similar to Row 3, Row 2 is similar to Row 4). To examine the phenomenon statistically, we conducted a mixed four-way repeated measures ANOVA with “the type of question: MW or DD” as the between-subject factor and “intentionality” (two levels: with intention and without intention), “duration” (two levels: short and long) as well as “type of self-generated thought” (five levels: recalling past events, fantasizing, planning, worrying, and ruminating) as the within-subject factor. Indeed, there was no significant interaction between “the type of question: MW or DD” and “intentionality: F(1,128) < 1”. Given that the average age of the participants was slightly higher in the DD group than in the MW group, we conducted a control analysis by including participants’ ages as a regressor. There was also no significant interaction between the type of question (MW or DD) and intentionality: F(1,127) < 1. Since intentionality of the self-generated thought did not affect whether the protagonist was perceived as mind-wandering or day-dreaming, we collapsed the data across intentionality for both short and long durations (i.e., short duration: averaging Rows 1 and 3 in Fig. 6; long duration: averaging Rows 2 and 4 in Fig. 6).

**Figure 7:**
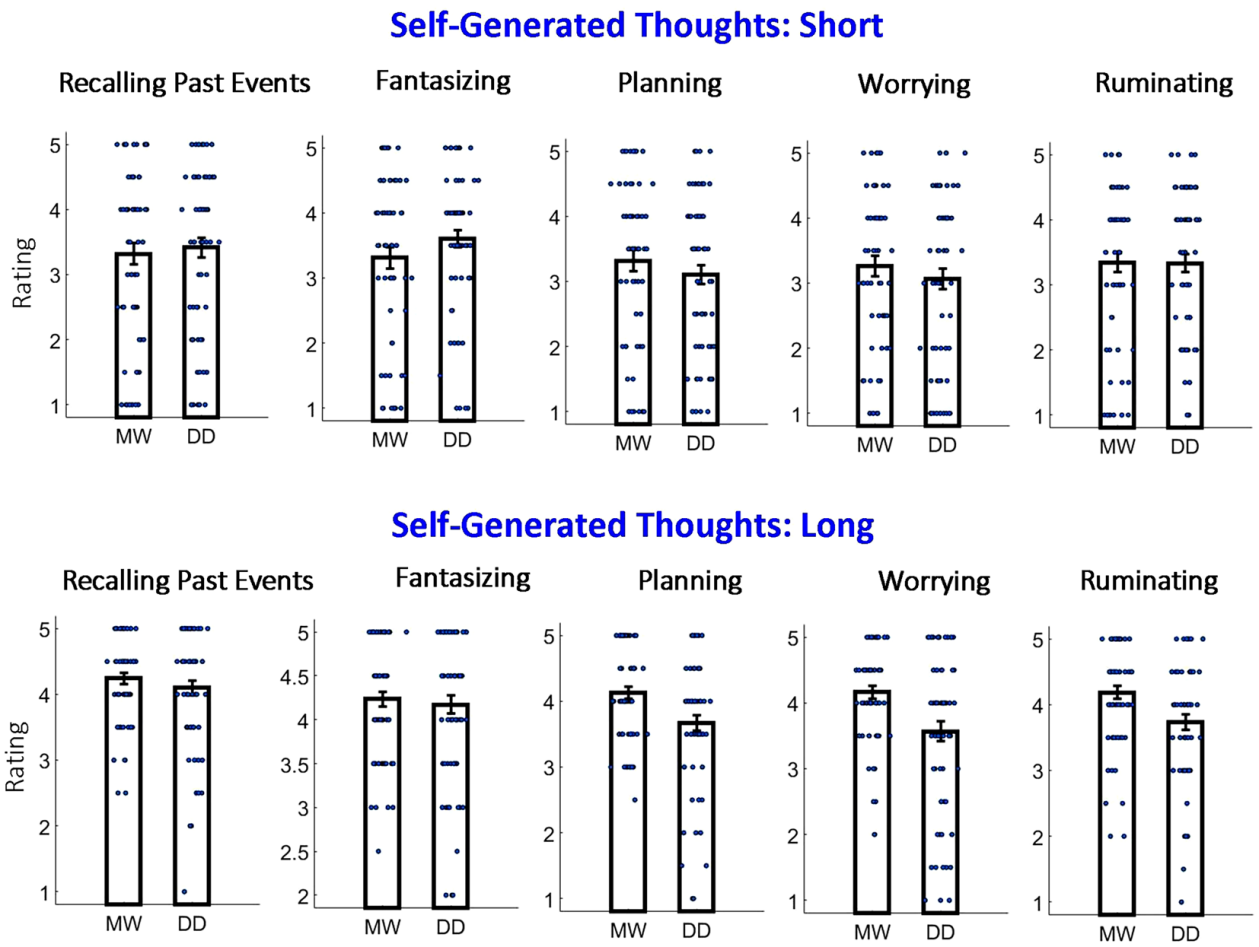
Results of Experiment 2 for situations of “short” or “long” internally directed thoughts (after collapsing “with intention” and “without intention”). The same convention used as in Figs. 3, 4, and 6. Note that the relationship between mind-wandering and day-dreaming differed between self-generated types of thought, but only for the “long” duration. Also, the relationship between mind-wandering and day-dreaming slightly (but without reaching significance) differed between “short” and “long”.

The results collapsed across intentionality are shown in Fig. 7. We can see some difference between patterns of the results for short and long duration with regard to the relationship between mind-wandering and day-dreaming. To test this statistically, we conducted a mixed three-way repeated-measures ANOVA with “the type of question: MW or DD” as the between-subject factor and “duration: short/long” as well as “type of self-generated thought” as within-subject factors. Indeed, an interaction between “the type of question: MW or DD” and “duration: short/long” was relatively high, but did not reach the statistical significance: F(1,128) = 2.64, p = 0.107, η^2^ = 0.006; control analysis with participants’ ages as the regressor: F(1,127) = 4.04, p = 0.046, η^2^ = 0.009. Our analysis also revealed a significant interaction between “the type of question: MW or DD” and “type of self-generated thought”: F(4,512) = 6.59, p < 0.001, η^2^ = 0.007; control analysis with participants’ ages as the regressor: F(1,127) = 5.44, p < 0.001, η^2^ = 0.007. This result suggests that the type of self-generated thought of a protagonist within the situation influenced whether the mental activity was considered mind-wandering or day-dreaming. Post-hoc *t*-test comparing mind-wandering and day-dreaming ratings revealed significantly higher mind-wandering ratings (after multiple comparison correction, n = 10; alpha = 0.05/10 = 0.005) for the long duration in “worrying” [t(128) = 3.37; p = 0.001; Cohen's d = 0.59], “rumination” [t(128) = 3.25; p = 0.003; Cohen's d = 0.53] and “planning” [t(128) = 3.02; p = 0.003; Cohen's d = 0.53].

### Replication experiments

To validate the reproducibility of our results, we repeated Experiments 1 and 2 using a sample size comparable to those in our main experiments. The full results of these experiments are reported in the Supplementary Material section (Supplementary Results and Supplementary Figs. 1–3). Critically, we fully replicated the phenomena reported in the main text. Specifically, we found that the situations were perceived differently with regard to mind-wandering and day-dreaming, depending on whether self-generated thoughts occurred when the participant was busy with another activity (Supplementary Fig. 2). The type of self-generated thought influenced whether the situation was perceived as mind-wandering or day-dreaming. In particular, “planning,” “worrying,” and “ruminative” thoughts caused the situation to be perceived more as mind-wandering, whereas “recalling past events” and “fantasizing” thoughts were more perceived to be day-dreaming (Supplementary Figs. 2 and 3). Duration of the self-generated thinking also affected whether the situation was perceived as mind-wandering or day-dreaming, but as with the main text, the effect was not strong (Supplementary Fig. 3). Interestingly, in the replication experiment, we found one effect that was not revealed in the main text experiments. In particular, we found that intentionality of the self-generated thought influenced as to whether the situation was perceived as mind-wandering or day-dreaming (Supplementary Fig. 3), but the effect was not strong.

### Exploring the reasons the situations were perceived as mind-wandering or day-dreaming

**Figure 8:**
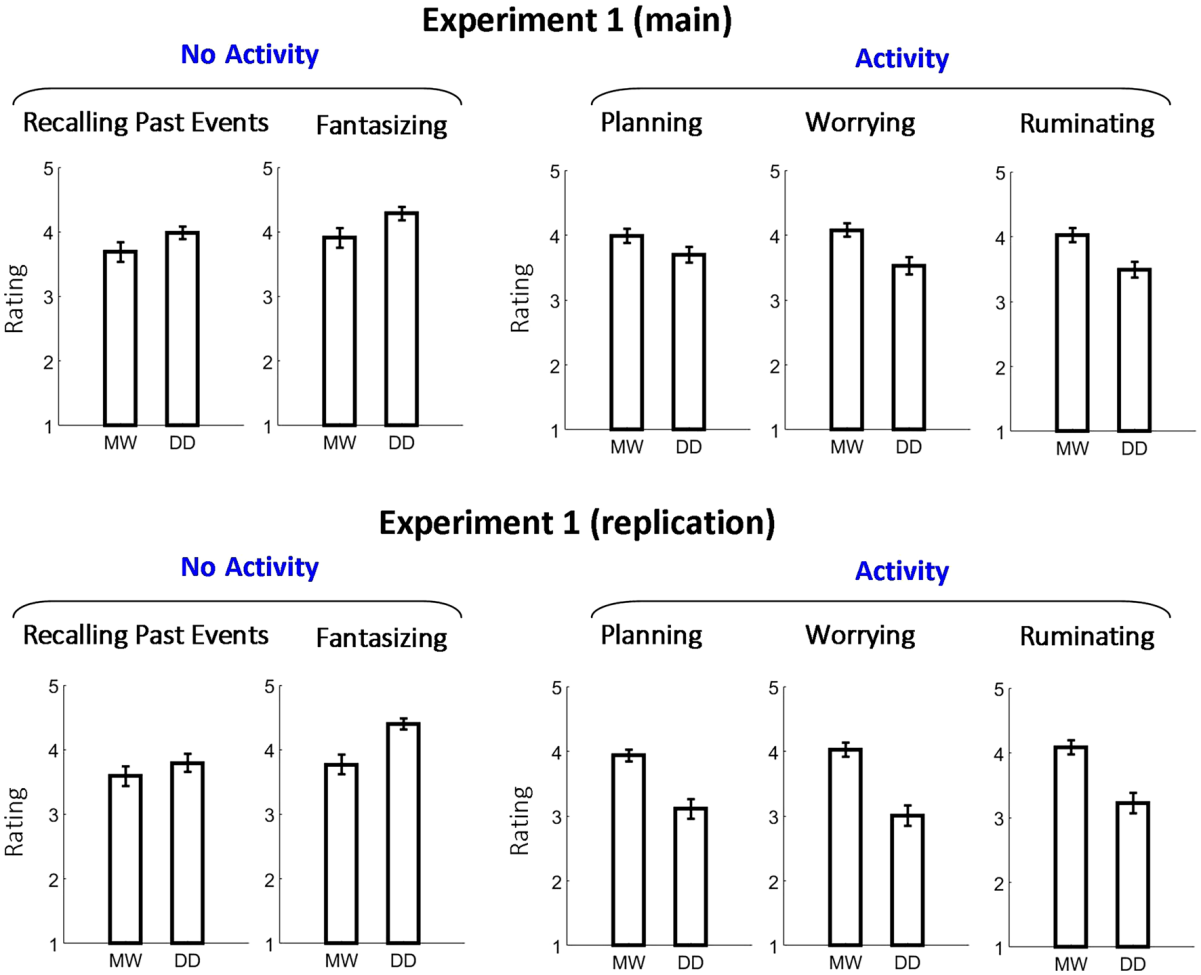
Results of Experiment 1 (main and replication experiments) for “planning,” “worrying,” and “ruminating” during another activity and “recalling past episodes” and “fantasizing” without another activity. The results showed a clear dissociation between the situations with planning, worrying, and ruminating thoughts (which were perceived more as mind-wandering) and the situations with recalling past episodes and fantasizing thoughts (which were perceived more as day-dreaming).

**Figure 9:**
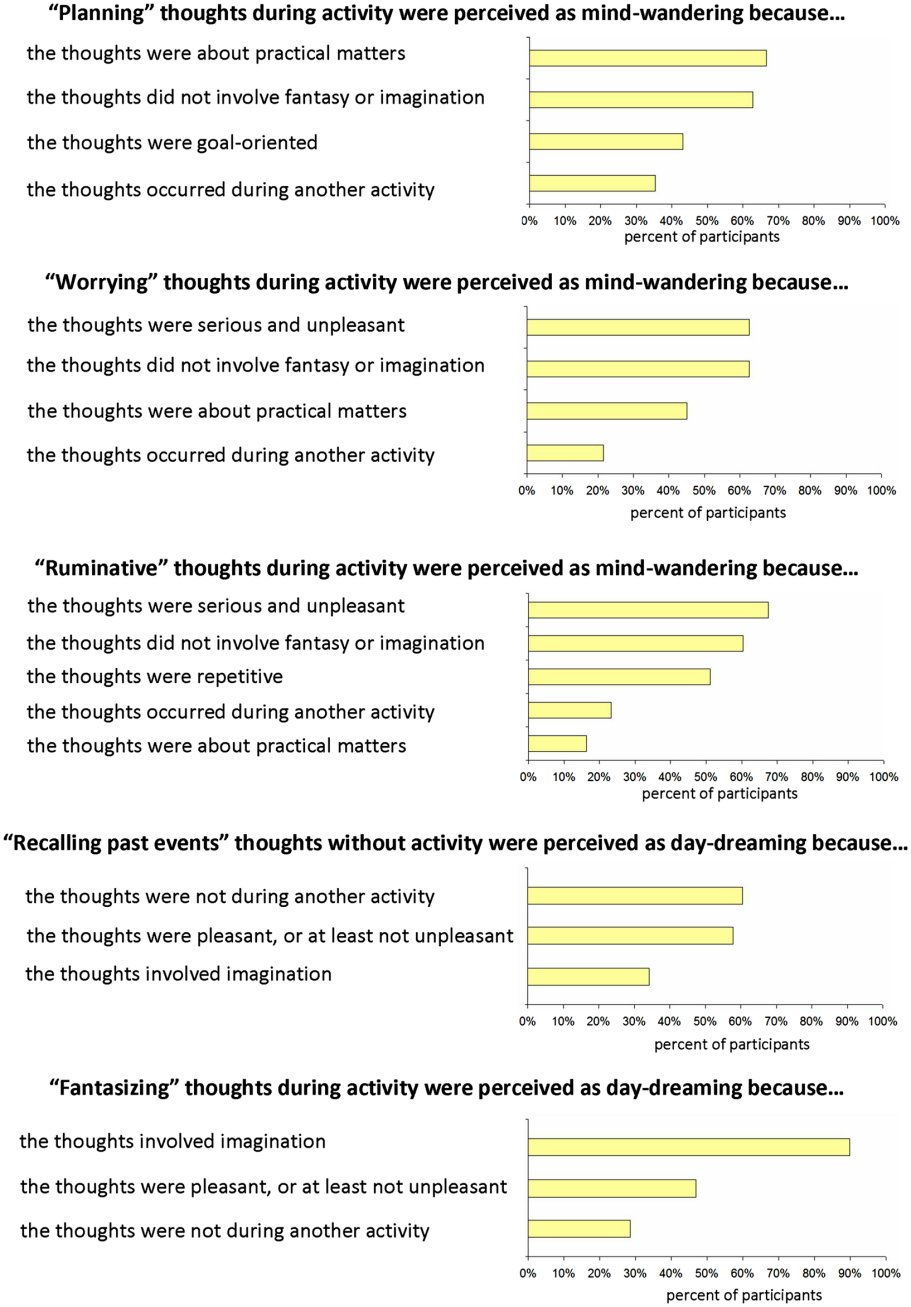
The reasons the participants classified the situations of planning, worrying, and ruminating during another activity as mind-wandering and recalling past episodes and fantasizing without another activity as day-dreaming. The participants could indicate more than one reason for each situation. Note that factor importance varied across different types of thought.

The strongest finding of our study was that planning, worrying, and ruminating self-generated thoughts, especially during another activity, tended to be perceived as mind-wandering, whereas recalling past episodes and fantasizing self-generated thoughts, especially without another activity, tended to be perceived as day-dreaming. In Fig. 8 we show the corresponding results of Experiment 1—both main and replication experiments. The pertinent question is why certain self-generated thoughts were perceived as mind-wandering while others were perceived as day-dreaming. To address this, in our final experiment a new group of participants was presented with five situations from Experiment 1 (planning, worrying, and ruminating thoughts during another activity and recalling past episodes and fantasizing thoughts without another activity). The participants were first asked whether it is more likely that the person in the situation mind-wandered or day-dreamed. Subsequently, the participants were asked to provide reasons for their answer. The participants answered that the situations with another activity was more likely mind-wandering than day-dreaming for planning (87.9%), worrying (87.9%) and ruminating (74.1%). In contrast, the participants answered that the situations without another activity were more likely a day-dreaming than mind-wandering for recalling past episodes (65.5%) and fantasizing (84.4%). These results provide important corroboration of our results obtained previously (Fig. 8). The reasons for participants classifying the situations are presented in Fig. 9. A common dominant reason to classify planning, worrying, and ruminating during an external activity as mind-wandering was the absence of fantasy or imagination in the self-generated thoughts. Additional dominant reasons were as follows: to classify planning as mind-wandering because the thoughts concerned practical matters, and to classify worrying and ruminating as mind-wandering because the thoughts were serious and unpleasant. In contrast, for both the recalling past events and fantasizing without external activity, an important reason to classify the thoughts as day-dreaming was because the thoughts were pleasant, or at least not unpleasant. In addition, the most dominant reason why fantasizing without external activity was classified as day-dreaming was that the thoughts involved the imagination.

## Discussion

Contemporary scientific literature usually treats mind-wandering and day-dreaming as synonymous (e.g.^6,10,27,34^), but are they? Surprisingly, to the best of our knowledge, there has been no study that has yet tested this empirically. Here, we tested to what extent the laypeople consider the terms mind-wandering and day-dreaming to be synonyms. In two experiments conducted twice (the main set and the replication set), participants evaluated the extent of mind-wandering and day-dreaming of a protagonist in different scenarios. We found that the situations were perceived differently with regard to mind-wandering and day-dreaming depending on whether self-generated thoughts occurred when the protagonist was busy with another activity and depending on the type (i.e., content) of the self-generated thoughts. In particular, across both experiments, in situations with another activity/task, the protagonist was perceived more as mind-wandering than day-dreaming in the case of planning, worrying, and ruminating thoughts (TUTs). In contrast, recalling past episodes and fantasizing self-generated thoughts, especially without another activity, tended to be perceived as day-dreaming. The duration of self-generated thinking had a relatively minor effect on whether the protagonist was perceived to be mind-wandering or day-dreaming, and intention of self-generated thinking had no consistent effect. Finally, we showed laypeople’s reasons for classifying the situation as mind-wandering or day-dreaming. Below we discuss the results in detail.

### Situations with different types of activity and MW/DD judgment

The most explored type of self-generated thoughts is task-unrelated thoughts (TUTs), which happen when instead of focusing on a primary task or activity, a person becomes engaged in self-generated thoughts. Studies that explore TUTs employ different tasks such as reading^55–57^, monotonous, sustained attention tasks^14,58,59^ and scene perception^60^, but usually only a single task is tested within one study (but see^61^). To increase the generalizability of our results, in our design we included two types of situations with activity: in one of them, the protagonist attended a meeting, lecture, or class, and in the other, the protagonist was busy with a task such as doing homework. This design permitted us to investigate both the activity with relatively passive engagement (i.e., attending a meeting, lecture, or class) and the activity with relatively active engagement (i.e., doing homework). Despite the differences between the two types of activity, the relationship between mind-wandering and day-dreaming was very similar for both types (Fig. 3 and Supplementary Fig. 1), suggesting that the way we perceive mind-wandering and day-dreaming might not depend on which specific activity the person is engaged in during TUTs.

### Presence of activity in situation and MW/DD judgment

The exploration of self-generated thoughts during a task (i.e., TUTs) is a convenient experimental approach, but self-generated thoughts happen not only during tasks or other activities but also, for example, during downtime^6^. To what extent the properties of self-generated thoughts during activity and without activity (e.g., downtime) differ is not well understood, but it is clearly important to distinguish between different situations of self-generated thinking in order to avoid theoretical complications^43^. Here, we found that whether a protagonist in the situation was engaged in the activity (i.e., self-generated thoughts were TUTs) or was not engaged in the activity (i.e., self-generated thoughts were during downtime) impacted whether the protagonist was perceived as mind-wandering or day-dreaming (Fig. 4 and Supplementary Fig. 2). In other words, the same self-generated thoughts were perceived more as mind-wandering or day-dreaming, depending on whether they happened during another activity. Thus, terminologically, the self-generated thoughts during another activity and the self-generated thoughts without another activity might not be fully equivalent. More specific differences between mind-wandering and day-dreaming are discussed further in the text.

### Duration of self-generated thoughts and MW/DD judgment

Based on our daily life experiences, self-generated thinking may be brief or may continue for a long time. For example, when a student attends a boring lecture, they might intentionally be absorbed in their own thoughts for many minutes. Interestingly, the duration and its impact on various properties of self-generated thinking has not been widely investigated. Here, we were interested in whether the duration of self-generated thinking influenced whether a situation was perceived as mind-wandering rather than day-dreaming. We used very short (seconds) and very long (20-min) durations to maximize the potential difference between the two conditions. In Experiment 2, which was conducted only for situations with self-generated thinking during an activity (i.e., TUTs), we found that the duration of TUTs had a relatively small impact (insignificant in the main experiment and significant in the replication experiment) on whether the situation was perceived as mind-wandering or day-dreaming (Fig. 7) and the differences between mind-wandering than day-dreaming ratings were larger in “long” duration. Specifically, in “long” duration the situation was perceived more as mind-wandering than day-dreaming in planning, worrying, and rumination (this is further discussed in section “Different types of self-generated thoughts and MW/DD judgment”).

### Intentionality of self-generated thoughts and MW/DD judgment

The intentionality of self-generated thinking has been actively explored, particularly in recent years^25^, showing that intentional and unintentional self-generated thinking might have different properties—as is the case, for example, when participants perform more or less effortful tasks^62^. In addition, intentional self-generated thinking is more future-oriented and less vague than unintentional thinking^63^. Accordingly, we were interested in testing whether intentionality of TUT influences whether the situation would be perceived as mind-wandering or day-dreaming. Our results were not conclusive; while in the main experiment we found that TUTs with and without intention were classified similarly with regard to mind-wandering and day-dreaming, in a replication experiment we found a significant ANOVA interaction between “the type of question: MW or DD” and intentionality, even if the effect size was relatively small. Thus, our overall results do not provide sufficiently clear evidence to draw firm conclusions.

### Different types of self-generated thoughts and MW/DD judgment

As we know from our daily lives, self-generated thoughts vary substantially by type and content. The seminal research program of Singer, Antrobus, Giambra, and colleagues launched more than half-a-century ago^5,64–68^, has established that self-generated thoughts (or day-dreaming) can be broadly subdivided into three types: positive-constructive day-dreaming, guilty-dysphoric day-dreaming (i.e., rumination), and poor attentional control. Phenomenological properties of self-generated thoughts have been actively investigated during recent years as well (for review:^10^). So, an important aspect of our work has been to understand the extent to which a person is perceived as mind-wandering or day-dreaming, depending on the content of their self-generated thoughts. The five types of self-generated thoughts that we tested broadly encompass different potential self-generated thoughts that have been widely investigated: recalling past events^7–10,69^, fantasizing^3,14–16^, future planning^8,9,17–20^ and worrying and ruminative repetitive thoughts^21–24^.

For both situations during activity (Fig. 4, top; Fig. 7; Supplementary Fig. 2; Supplementary Fig. 3) and without activity (Fig. 4, bottom; Supplementary Fig. 2, bottom), we found that the type of self-generated thoughts influenced whether the thoughts were classified as mind-wandering or day-dreaming (i.e., a significant ANOVA interaction between the type of question [MW or DD] and the type of self-generated thought). With regard to specific types of thought, we found that in situations involving another activity/task (TUTs), the protagonist was perceived as mind-wandering rather than day-dreaming in the case of planning, worrying, or ruminating thoughts. In contrast, for recalling past events and fantasizing, the protagonist was perceived more as day-dreaming than mind-wandering, particularly when there was no external activity (Fig. 8). Our follow-up investigation established that for the thought to be classified by lay participants as mind-wandering, it is particularly important that it lacks fantasy and imagination (planning, worrying, and ruminating), is focused on practical matters (planning), and is serious and potentially unpleasant (worrying and ruminating). By contrast, for thoughts to be classified as day-dreaming, it is particularly important that they not occur during another activity and that they are positive or at least neutral (recalling past events and fantasizing) and involve imagination (fantasizing). These results demonstrate an interesting dissociation as to how laypeople perceive mind-wandering and day-dreaming terms. That is, while mind-wandering can be associated with negative concrete thoughts that can be a distraction from another activity, day-dreaming is more associated with flights of fancy and more positive thoughts that occur when there is no another activity. Interestingly, the valence of the self-generated thoughts (i.e., negative vs. positive) seems to be one of the important factors in distinguishing between mind-wandering and day-dreaming episodes. While the valence of the self-generated thoughts has been explored previously^2,70,71^, the distinction between mind-wandering and day-dreaming based on valence has not usually been emphasized. Overall, we think that in light of ongoing terminological debate in the field of mind-wandering^41–44^, the dissociation between mind-wandering and day-dreaming as perceived by laypeople might bring us closer to understanding the phenomenon.

### Practical implications

To date, while some questionnaires like the Daydreaming Frequency Scale^37^ or NEO Personality Inventory^38^ use the term “day-dreaming,” other questionnaires like the Mind-Wandering Questionnaire^39^ and Deliberate and Spontaneous Mind-wandering Scale^40^ use the term “mind-wandering.” Notably, in some cases, the terms are used without any further explanations, like for example NEO Personality Inventory questionnaire item: “I don't like to waste my time daydreaming”^38^. But as we demonstrated, the mere fact of which term is used might bias the result. We thus suggest that future questionnaires, regardless of the term they use, provide a sufficiently detailed explanation of what is meant by the term. This will facilitate interpretation and comparison of the results of different studies.

### Limitations and potential concerns

Self-generated thought is a heterogeneous phenomenon, so its proper terminological definition (e.g., mind-wandering, day-dreaming, task- or stimulus-related thoughts) has been debated. According to the recently proposed family resemblances view^43^, mind-wandering can function as an umbrella term while emphasizing different characteristics depending on the specific experimental situation/manipulation/condition. In light of this proposal, the present investigation might seem to be of limited use, but as we explain below, we do not think that this is case. First, not all researchers have endorsed the proposal of Seli and colleagues. In particular, Christoff and colleagues^42^ argue that adoption of the family resemblances approach might be problematic because instead of trying to identify specific features of mind-wandering, the use of mind-wandering as an umbrella term can lead to overgeneralization. Accordingly, illustrating the difference between mind-wandering and day-dreaming, as we showed here, is still important for terminological debate. Second, even if we adopt the approach of Seli and colleagues, it is still important to properly characterize the mind-wandering phenomenon^43^. We therefore show under what conditions and why self-generated thought will tend to be perceived as day-dreaming. Finally, as we emphasized in the “Practical Implications” section, research questionnaires have used the terms mind-wandering and day-dreaming interchangeably. Our results show that mind-wandering and day-dreaming terms are not synonymous, potentially aiding in the better interpretation of previous findings. All this emphasizes the potential value and impact of our findings.

A notable aspect of our results was that even for situations in which we obtained significant differences between mind-wandering and day-dreaming, the average ratings for both types of self-generated thoughts were relatively high (about 3 and above). For example, while the scores of situations with “planning” thoughts during activity were higher for mind-wandering than day-dreaming, the average day-dreaming scores were about 3–3.5 (depending on the experiment). How can such results be interpreted? First, we should take into account that we are dealing with average scores, so some participants did not think that planning thoughts during an activity was day-dreaming (i.e., gave low scores). Second, the participants’ ratings could be also influenced by an implicit baseline with which the situations used in the experiment were compared. That is, all our scenarios included a protagonist engaged with self-generated thoughts. Compared to activities like playing basketball or talking with a friend, any scenario with self-generated thoughts might be considered at least to some extent mind-wandering or day-dreaming. This may explain why the obtained scores were relatively high. Critically, the main goal of our study was to examine the relationship between mind-wandering and day-dreaming scores, and the absolute scores were therefore of less interest in the present study.

In the present study the participants were recruited via the Amazon Mechanical Turk (mTurk) platform. The major added value of the mTurk platform is that it simplifies experimental procedures and yields a large pool of accessible participants (close to 1,000 participants in our case), permitting sufficient statistical power. In addition, our specific study required participants to be native English speakers, but finding a large number of such participants in a non-English speaking country (Israel, in our case) would have been challenging. Having said that, when the study is conducted using mTurk platform, one should be aware of potential concerns and limitations^72^. Specifically, the researcher should ensure that the participants are sufficiently attentive and speak the appropriate native language (English, in our case). An additional potential concern is that mTurk participants take part in many studies and therefore might not be naive, so their responses might differ from naive participants. To address potential concerns about inattention, in our questionnaire we included “bogus” items of different types^50,52,53^. These items were not only of the arithmetic type (which easily pop up out of other items), but also common knowledge items that appeared visually similar to the items of interest. At the data analysis stage, we rigorously excluded participants who made errors with the “bogus” items. In addition, the fact that we fully replicated our results in two additional experiments also inspires confidence in the resulting effects. To ensure that English was the native language of the participants, we restricted our experiment to the US and also excluded participants with non-English first languages. Finally, as we explain below, we do not think that potential lack of naivety of the participants had a major impact on our results. The most serious concern regarding the participants naivety is whether they have previously taken part in the same or a similar experiment. The concern is amplified with experiments that include deception^73^ or if participants learn the incentive structure^74^. Critically, this was not the case with our study because the questionnaires were designed by us and have not been used previously, so the participants could not have any prior exposure to them. Even if the participants had answered another questionnaire on mind-wandering or day-dreaming, in contrast to the experiments involving deception or an incentive structure, it seems very unlikely that this could influence their answers in our experiment. Finally, in our data analysis we mainly focused on comparing the MW and DD groups of participants, so if any prior bias existed (and we do not think that it was the case), the bias would have appeared in both groups. Nevertheless, it would be interesting to conduct a similar study in a non-online format in the future.

## Conclusion

In the present online study, we asked the participants to evaluate to what extent a protagonist mind-wanders or day-dreams in different scenarios. We demonstrated cases with divergent results with regard to mind-wandering and day-dreaming judgment, suggesting that the two terms might not be fully equivalent. We also demonstrated why laypeople tend to label the phenomenon mind-wandering or day-dreaming. As we explained in the “Discussion”, these results have both theoretical and practical implications.

### Supplementary Information

Supplementary Information.

## Data Availability

The data can be accessed via link: https://github.com/vax2023/MW_DD_paper_raw_data.
