# Supplementary material for: Elucidating the difference between mind-wandering and day-dreaming terms
Source: Sci Rep. 2024 May 21;14:11598. doi: 10.1038/s41598-024-62383-7 (PMC11109208; doi:10.1038/s41598-024-62383-7)
Supplement: Supplementary file 1 — Supplementary Information. [file 41598_2024_62383_MOESM1_ESM.pdf]

Supplementary Materials

Contents

Supplementary Results .... pages 2 − 4

Supplementary Figures .... pages 5 − 7

Supplementary Tables .... pages 8 − 11

Supplementary Results

3.1 Experiment 1

As in the main text, first, in our analysis, we focused on the situations in which a protagonist engaged in two types of activity (i.e., attending a meeting/lecture/class or being busy with a task, like doing homework). These results are shown in Supplementary Fig. 1. A mixed three-way ANOVA with "the type of question: mind-wandering (MW) or day-dreaming (DD) as the between-subject factor and "type of activity" (two levels: attending a lecture/meeting/class or doing a task like homework) as well as "type of self-generated thought" (five levels: recalling past events, fantasizing, planning, worrying, and ruminating) as within-subject factors revealed no significant two-way interaction between "the type of question: MW or DD" and "type of activity" [$F(1,122) < 1$]. Since there was no difference between the types of activity (i.e., attending a meeting/lecture/class and engaging in a task like homework) with regard to mind-wandering and day-dreaming, we collapsed the data into one category (i.e., "Activity").

The results of the "Activity" and "No Activity" (i.e., downtime) situations across five types of self-generated thought are shown in Supplementary Fig. 2. To examine the effects, we conducted a mixed three-way repeated-measures ANOVA with "the type of question: MW or DD" as the between-subject factor and "activity: yes/no" as well as "type of self-generated thought" as within-subject factors. We found a significant interaction between "the type of question: MW or DD" and "activity yes/no" [$F(1,129) = 12.7$, $p < 0.001$, $\eta^2 = 0.013$], suggesting that whether a protagonist in the situation was engaged in the activity influenced whether self-generated thought was perceived as mind-wandering or day-dreaming. Additionally, we found a significant interaction between "the type of question: MW or DD" and "type of self-generated thought" [$F(4,488) = 13.57$, $p < 0.001$, $\eta^2 = 0.029$], suggesting that the type of self-generated thought influenced whether the protagonist's mental activity was considered mind-wandering or day-dreaming.

Given that the presence of activity influenced whether self-generated thought was perceived as mind-wandering or day-dreaming, we conducted two separate analyses for "Activity" and "No Activity." For the "Activity," two-way repeated-measure ANOVAs with "the type of question: MW or DD" as the between-subject factor and

"the type of self-generated thought" as the within-subject factor revealed significant main effects of "the type of question: MW or DD" [$F(1,122) = 25.07$, $p < 0.001$, $\eta^2 = 0.091$] and "the type of self-generated thought" [$F(4,488) = 30.28$, $p < 0.001$, $\eta^2 = 0.087$], as well as significant interaction between "the type of question: MW or DD" and "the type of self-generated thought" [$F(4,488) = 9.99$, $p < 0.001$, $\eta^2 = 0.029$]. The post-hoc t-test comparing mind-wandering and day-dreaming ratings revealed significantly higher mind-wandering ratings (after multiple comparison correction, n=5; alpha = 0.05/5 = 0.01) in "worrying" [$t(122)=3.29$; $p < 0.001$; Cohen's $d = 0.94$], "planning" [$t(122)=4.61$; $p < 0.001$; Cohen's $d = 0.83$] and "rumination" [$t(122)=4.48$; $p < 0.001$; Cohen's $d = 0.81$]. Mind-wandering compared to day-dreaming ratings were also higher in "recalling past events" [$t(122)=2.68$; $p = 0.008$; Cohen's $d = 0.48$], albeit the difference was much smaller compared to the "worrying", "planning" and "rumination"

For the "No Activity," two-way repeated-measures ANOVAs with "the type of question: MW or DD" as the between-subject factor and "the type of self-generated thought" as the within-subject factor did not reveal significant main effects of "the type of question: MW or DD" [$F(1,122) <1$], revealed a significant main effect of "the type of self-generated thought" [$F(4,488) = 24.13$, $p < 0.001$, $\eta^2 = 0.066$], as well as a significant interaction between "the type of question: MW or DD" and "the type of self-generated thought" [$F(4, 488) = 11.4$, $p < 0.001$, $\eta^2 = 0.031$]. The post-hoc t-test comparing day-dreaming and mind-wandering ratings revealed higher (after the multiple comparison correction, n=5; alpha = 0.05/5 = 0.01) day-dreaming ratings in "fantasizing" [$t(122)=3.64$; $p < 0.001$; Cohen's $d = 0.65$]. In addition, mind-wandering compared to day-dreaming ratings were higher in "worrying", albeit without reaching significance multiple comparison correction [$t(122)=2.57$; $p = 0.011$; Cohen's $d = 0.46$].

<u>3.2 Experiment 2</u>

The results for all conditions are shown in Supplementary Fig. 3. As in the main text, we conducted a mixed four-way repeated measures ANOVA with "the type of question: MW or DD" as the between-subject factor and "intentionality" (two levels: with intention and without intention), "duration" (two levels: short and long) as well as "type of self-generated thought" (five levels: recalling past events, fantasizing,

planning, worrying, and ruminating) as the within-subject factor. We found significant, albeit not a strong, interactions between "the type of question: MW or DD" and "intentionality" [$F(1,127) = 5.818$, $p = 0.017$, $\eta^2 = 0.005$] and between "the type of question: MW or DD" and "duration" [$F(1,127) = 4.7$, $p = 0.032$, $\eta^2 = 0.007$]. In addition, there was a relatively strong interactions between "the type of question: MW or DD" and "type of self-generated thought" [$F(1,508) = 14.192$, $p < 0.001$, $\eta^2 = 0.017$]. As the "the type of question: MW or DD" / "intentionality" and "the type of question: MW or DD" / "duration" interactions were significant, we did not collapse the results further.

Post-hoc t-test comparing mind-wandering and day-dreaming ratings revealed significantly higher mind-wandering ratings (after multiple comparison correction, n=20; alpha = 0.05/20 = 0.0025) for the long duration / no intention in "worrying" [$t(127)=5.48$; $p < 0.001$; Cohen's $d = 0.96$] and long duration / no intention "rumination" [$t(127)=3.91$; $p < 0.001$; Cohen's $d = 0.68$]. In addition, mind-wandering ratings were higher, but without reaching significance after multiple comparison correction for long duration / no intention "planning" [$t(127)=2.95$; $p = 0.004$; Cohen's $d = 0.52$], long duration / intention "worrying" [$t(127)=2.71$; $p = 0.008$; Cohen's $d = 0.48$] and duration / intention "rumination" [$t(127)=2.1$; $p = 0.038$; Cohen's $d = 0.37$]. In contrast, day-dreaming ratings were higher, but without reaching significance after multiple comparison correction for short duration / intention "recalling past events" [$t(127)=3.02$; $p = 0.003$; Cohen's $d = 0.53$], short duration / intention "fantasizing" [$t(127)=3.01$; $p = 0.003$; Cohen's $d = 0.53$] and long duration / intention "fantasizing" [$t(127)=2.92$; $p = 0.004$; Cohen's $d = 0.52$].

Supplementary Figures

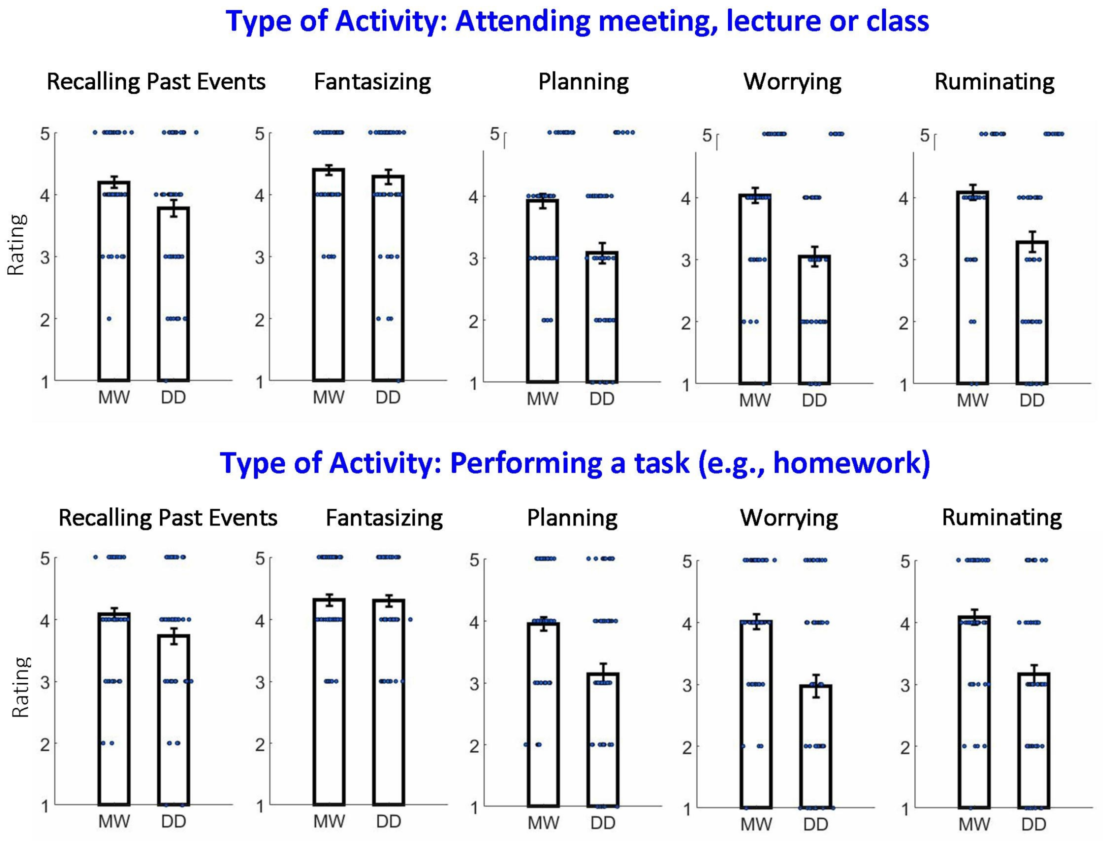

Supplementary Figure 1. Results of replication Experiment 1 for situations with two types of activities: attending a meeting/lecture/class or being busy with a task like doing homework. The same convention as in main text figures. Note that the two types of activity were similar with regard to the relationship between mind-wandering and day-dreaming. Note similarity of the results to the results of the main Experiment 1, see Figure 2.

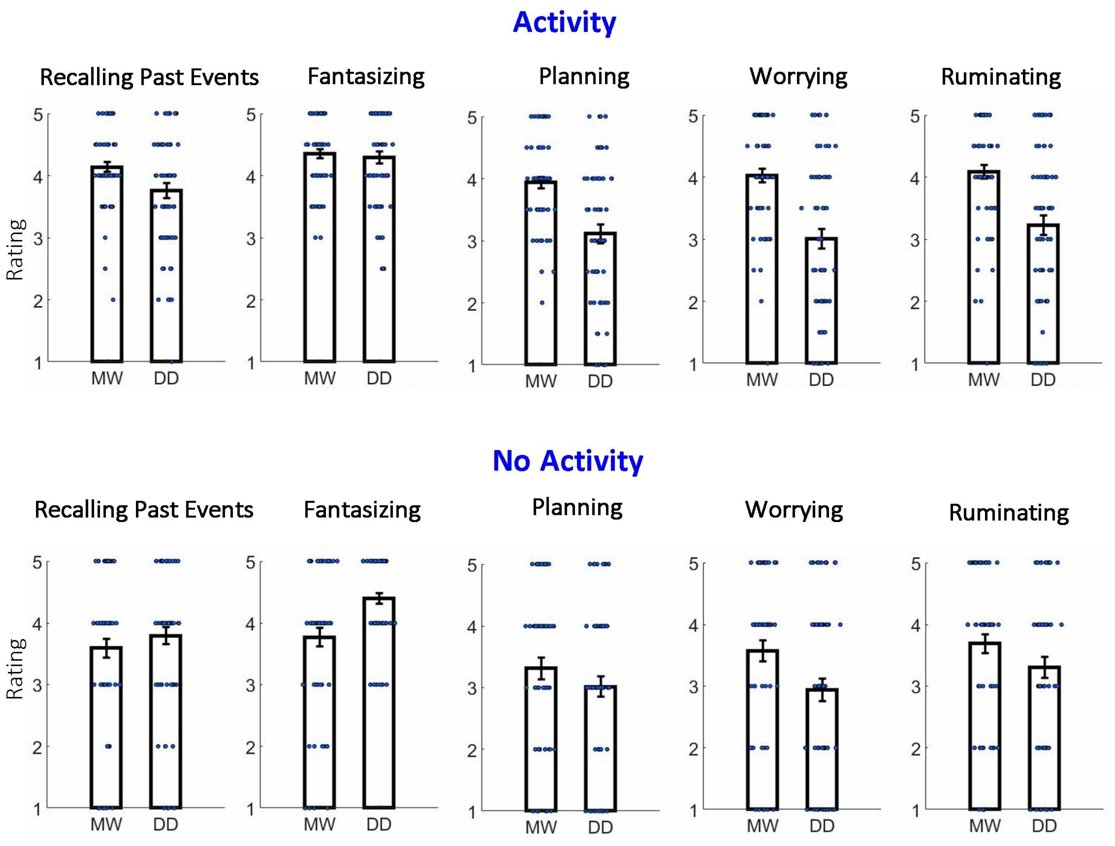

Supplementary Figure 2.. Results of replication Experiment 1 for situations with "Activity" (after collapsing attending a meeting/lecture/class or being busy with a task like doing homework) and "No Activity" (i.e., downtime). Note that on one hand the relationship between mind-wandering and day-dreaming differed between the "Activity" and "No Activity" situations and on other hand, the relationship between mind-wandering and day-dreaming differed between self-generated types of thought. Note similarity of the results to the results of the main Experiment 1, see Figure 3.

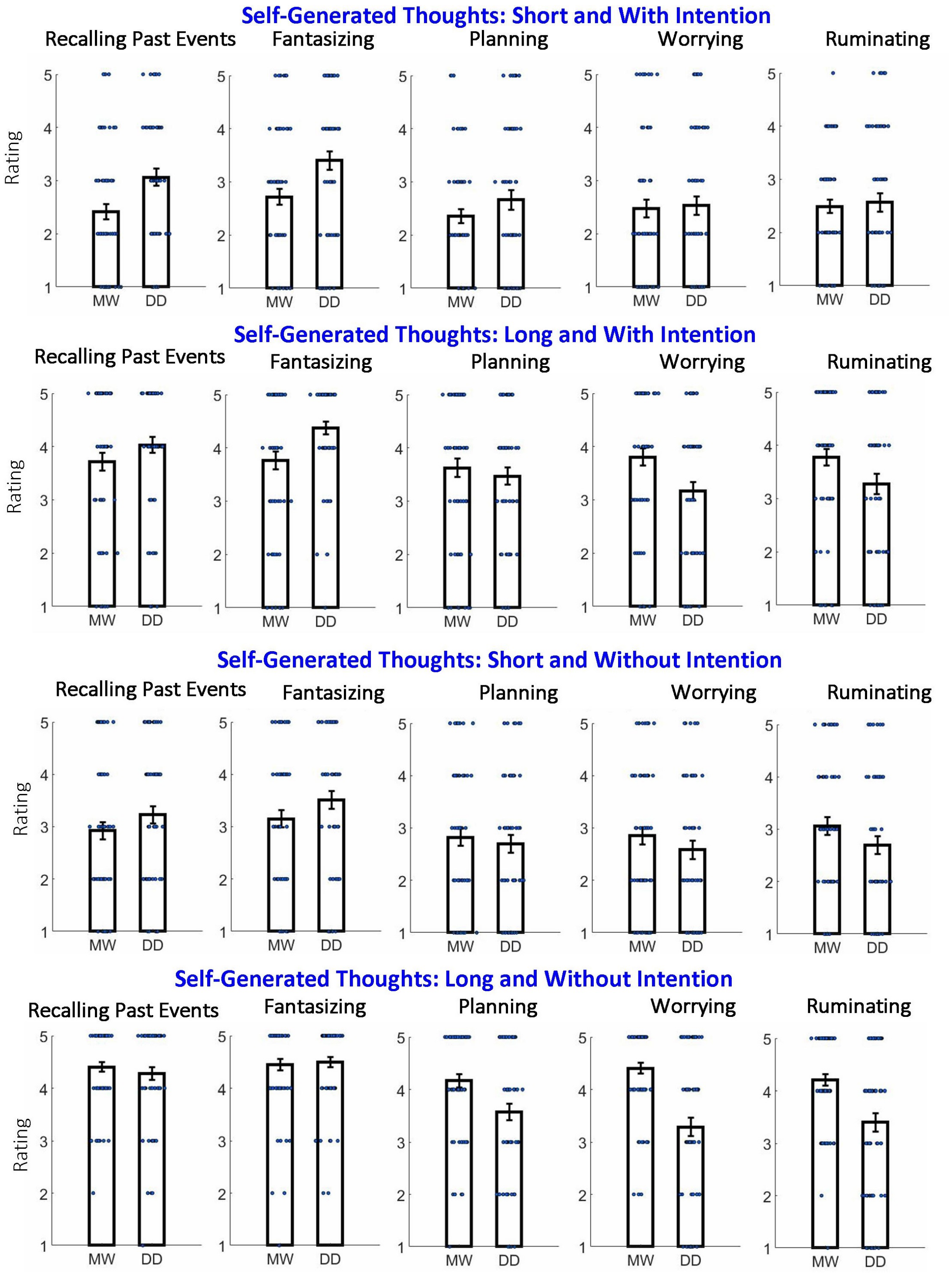

Supplementary Figure 3. Results of replication Experiment 2 for situations of either "short" or "long" and "with intention" or "without intention" internally directed thoughts. The same convention are used as in Figs. 3 and 4. Note that while for "planning", "worrying" and "rumination", particularly for "long" durations, the situations were perceived more as mind-wandering, for "recalling past events" and "fantasizing" the situations were perceived more as day-dreaming. Note similarity of the results to the results of the main Experiment 2, see Figure 6.

Supplementary Tables

ID	Type Of Activity	Type Of Internally Directed Thought	Description of the situation
1	Attending Meeting / Lecture / Class	Recalling Past Events	A person has been attending an important meeting, lecture or class, but at some point started to recall something unrelated from the past (e.g., yesterday's romantic date).
2	Attending Meeting / Lecture / Class	Fantasizing	A person has been attending an important meeting, lecture or class, but at some point started to fantasize about something unrelated (e.g., about a romantic date tomorrow).
3	Attending Meeting / Lecture / Class	Planning	A person has been attending an important meeting, lecture or class, but at some point, started to plan something unrelated (e.g., what he/she needs to buy in the supermarket).
4	Attending Meeting / Lecture / Class	Worrying	A person has been attending an important meeting, lecture or class, but at some point started to worry about something unrelated (e.g., the condition of his/her mother who is at the hospital).
5	Attending Meeting / Lecture / Class	Ruminating	A person has been attending an important meeting, lecture or class, but at some point he/she was stuck with unrelated repetitive negative thoughts (e.g., replaying over and over a recent failure).
6	Task (e.g., Homework)	Recalling Past Events	A person has been busy with a task (e.g., doing homework), but at some point started to recall something unrelated from the past (e.g., yesterday's romantic date).
7	Task (e.g., Homework)	Fantasizing	A person has been busy with a task (e.g., doing homework), but at some point started to fantasize about something unrelated (e.g., about a romantic date tomorrow).
8	Task (e.g., Homework)	Planning	A person has been busy with a task (e.g., doing homework), but at some point started to plan something unrelated (e.g., what he/she needs to buy in the supermarket).
9	Task (e.g., Homework)	Worrying	A person has been busy with a task (e.g., doing homework), but at some point started to worry about something unrelated (e.g., the condition of his/her mother who is at the hospital).
10	Task (e.g., Homework)	Ruminating	A person has been busy with a task (e.g., doing homework), but at some point he/she was stuck with unrelated repetitive negative thoughts (e.g., replaying over and over a recent failure)
11	No activity	Recalling Past Events	A person had some down-time (e.g., was waiting in a queue). During this time he/she was recalling a past event (e.g., yesterday's romantic date).
12	No activity	Fantasizing	A person had some down-time (e.g., was waiting in a queue). During this time he/she was fantasizing (e.g., about a romantic date tomorrow).

13	No activity	Planning	A person had some down-time (e.g., was waiting in a queue). During this time he/she was planning something (e.g., what he/she needs to buy in the supermarket).
14	No activity	Worrying	A person had some down-time (e.g., was waiting in a queue). During this time he/she was worrying (e.g., about the upcoming surgery of his/her mom).
15	No activity	Ruminating	A person had some down-time (e.g., was waiting in a queue). During this time he/she was stuck with unrelated repetitive negative thoughts (e.g., replaying over and over a recent failure)

Supplementary Table 1. Scenarios presented to participants in Experiment 1. The "ID" column (column 1) represents the question number. The "type of activity"(column 2) depicts what the person in the scenario is doing, for example, attending a meeting/lecture/class, being busy with a task (doing homework) and no activity (downtime like waiting in a queue). "Type of internally directed thought" (column 3) represents the internal experience of the person in the situation. Lastly, the "description of the situation" (column 4) is provided as the scenario depicted before the question.

ID	Type Of Internally Directed Thought	With / Without Intention	Duration	Description of the situation
1	Recalling Past Events	Intention	Short	A person has been attending an important meeting, lecture or class. At some point, just for several seconds, he/she intentionally disengaged from the event by recalling something unrelated from the past (e.g., yesterday's romantic date).
2	Fantasizing	Intention	Short	A person has been attending an important meeting, lecture or class. At some point, just for several seconds, he/she intentionally disengaged from the event by fantasizing about something unrelated (e.g., about a romantic date tomorrow).
3	Planning	Intention	Short	A person has been attending an important meeting, lecture or class. At some point, just for several seconds, he/she intentionally disengaged from the event by planning something unrelated (e.g., what he/she needs to buy in the supermarket).
4	Worrying	Intention	Short	A person has been attending an important meeting, lecture or class. At some point, just for several seconds, he/she intentionally disengaged from the event by worrying about something unrelated (e.g., the condition of his/her mother who is at the hospital).
5	Ruminating	Intention	Short	A person has been attending an important meeting, lecture or class. At some point, just for several seconds, he/she intentionally disengaged from the event by being stuck with unrelated repetitive negative thoughts (e.g., replaying over and over a recent failure).
6	Recalling Past Events	Intention	Long	A person has been attending an important meeting, lecture or class. At some point, for 20 minutes straight, he/she intentionally disengaged from the event by recalling something unrelated from the past (e.g., yesterday's romantic date).
7	Fantasizing	Intention	Long	A person has been attending an important meeting, lecture or class. At some point, for 20 minutes straight he/she intentionally disengaged from the event by fantasizing about something unrelated (e.g., about a romantic date tomorrow).
8	Planning	Intention	Long	A person has been attending an important meeting, lecture or class. At some point, for 20 minutes straight he/she intentionally disengaged from the event by planning something unrelated (e.g., what he/she needs to buy in the supermarket).
9	Worrying	Intention	Long	A person has been attending an important meeting, lecture or class. At some point, for 20 minutes straight he/she intentionally disengaged from the event by worrying about something unrelated (e.g., the condition of his/her mother who is at the hospital).
10	Ruminating	Intention	Long	A person has been attending an important meeting, lecture or class. At some point, for 20 minutes straight he/she intentionally disengaged from the event by being stuck with unrelated repetitive negative thoughts (e.g., replaying over and over a recent failure).
11	Recalling Past Events	No Intention	Short	A person has been attending an important meeting, lecture or class. At some point, just for several seconds, he/she without intention disengaged from the event by recalling something unrelated from the past (e.g., yesterday's romantic date).

12	Fantasizing	No Intention	Short	A person has been attending an important meeting, lecture or class. At some point, just for several seconds, he/she without intention disengaged from the event by fantasizing about something unrelated (e.g., about a romantic date tomorrow).
13	Planning	No Intention	Short	A person has been attending an important meeting, lecture or class. At some point, just for several seconds, he/she without intention disengaged from the event by planning something unrelated (e.g., what he/she needs to buy in the supermarket).
14	Worrying	No Intention	Short	A person has been attending an important meeting, lecture or class. At some point, just for several seconds, he/she without intention disengaged from the event by worrying about something unrelated (e.g., the condition of his/her mother who is at the hospital).
15	Ruminating	No Intention	Short	A person has been attending an important meeting, lecture or class. At some point, just for several seconds, he/she without intention disengaged from the event by being stuck with unrelated repetitive negative thoughts (e.g., replaying over and over a recent failure).
16	Recalling Past Events	No Intention	Long	A person has been attending an important meeting, lecture or class. At some point, for 20 minutes straight, he/she without intention disengaged from the event by recalling something unrelated from the past (e.g., yesterday's romantic date).
17	Fantasizing	No Intention	Long	A person has been attending an important meeting, lecture or class. At some point, for 20 minutes straight, he/she without intention disengaged from the event by fantasizing about something unrelated (e.g., about a romantic date tomorrow).
18	Planning	No Intention	Long	A person has been attending an important meeting, lecture or class. At some point, for 20 minutes straight, he/she without intention disengaged from the event by planning something unrelated (e.g., what he/she needs to buy in the supermarket).
19	Worrying	No Intention	Long	A person has been attending an important meeting, lecture or class. At some point, for 20 minutes straight, he/she without intention disengaged from the event by worrying about something unrelated (e.g., the condition of his/her mother who is at the hospital).
20	Ruminating	No Intention	Long	A person has been attending an important meeting, lecture or class. At some point, for 20 minutes straight, he/she without intention disengaged from the event by being stuck with unrelated repetitive negative thoughts (e.g., replaying over and over a recent failure).

Supplementary Table 2. Scenarios presented to participants in Experiment 2. The "ID" column (column 1) represents the question number. The "type of internally directed thought" (column 2) represents the internal experience of the person in the situation. "With / Without Intention" (column 3) represents whether the person in the scenario intentionally disengaged or not. The "duration" classified the scenarios to either short (several seconds) or long (20 minutes). Lastly, the "description of the situation" (column 5) is the description of the situation.
